# Immunomodulation by bacterial products promotes innate signatures favorable to macrophage responses in tuberculosis infection

**DOI:** 10.3389/fimmu.2025.1664444

**Published:** 2025-12-19

**Authors:** Dámaris P. Romero-Rodríguez, Eduardo Montes, Héctor Isaac Rocha-González, Martha Torres, Joaquín Zúñiga, Esmeralda Juárez

**Affiliations:** 1 Laboratorio Nacional Conahcyt de Investigación y Diagnóstico por Inmunocitofluorometría (LANCIDI), Instituto Nacional de Enfermedades Respiratorias Ismael Cosío Villegas, Ciudad de México, Mexico; 2 Sección de Estudios de Posgrado e Investigación, Escuela Superior de Medicina, Instituto Politécnico Nacional, Ciudad de México, Mexico; 3 Laboratorio de Biología Molecular, Instituto Nacional de Enfermedades Respiratorias Ismael Cosío Villegas, Ciudad de México, Mexico; 4 Laboratorio de Inmunobiología de la Tuberculosis, Instituto Nacional de Enfermedades Respiratorias Ismael Cosío Villegas, Ciudad de México, Mexico; 5 Laboratorio de Imunobiología y Genética, Instituto Nacional de Enfermedades Respiratorias Ismael Cosío Villegas, Ciudad de México, Mexico; 6 Tecnologico de Monterrey, Escuela de Medicina y Ciencias de la Salud, Mexico City, Mexico; 7 Laboratorio de Alta Contención Biológica (LACBio), Instituto Nacional de Enfermedades Respiratorias Ismael Cosío Villegas, Ciudad de México, Mexico

**Keywords:** bacterial lysates, bacterial suspension, macrophages, metabolic immunomodulation, trained immunity, tuberculosis

## Abstract

**Background:**

Tuberculosis remains a leading cause of death from infectious diseases globally, underscoring the need to boost innate responses in monocytes and macrophages to enhance early control of Mycobacterium tuberculosis infection. Trained immunity, a form of innate immune memory, enhances macrophage responsiveness through epigenet ic and metabolic reprogramming, offering a promising approach to strengthen host defenses against M. tuberculosis.

**Methods:**

This study evaluated the immunomodulatory potential of pharmaceutical-grade bacterial suspension (BS) and bacterial lysates (BL) in human monocyte-derived macrophages (MDM) and their role in innate response to M. tuberculosis infection. MDMs were stimulated with M. bovis BCG, BS, and BL following a training protocol described for BCG-dependent trained immunity.

**Results:**

We observed that BS and BL induced sustained cytokine responses and a metabolic transcriptional profile upon secondary stimulation with M. tuberculosis. BS and BL promoted increased IL-1b production in M. tuberculosis-infected MDMs. Additionally, the expression of surface markers shifted to high levels of CD80, CD86, HLA-DR, TLR2, and CD16 and low expression of CD163, TLR9, CCR2, and TLR4, consistent with an M1 phenotype. Moreover, BS and BL upregulated antimicrobial transcriptional signatures, including autophagy-related MAP1LC3 and ATG16L1.

**Discussion:**

These findings indicate that BS and BL engage training-associated transcriptional and phenotypic changes, providing new adjunctive strategies to boost innate responses in tuberculosis and other chronic infections.

## 1 Introduction

Despite widespread use of antibiotics and the BCG vaccine, tuberculosis (TB) remains a leading cause of death from infectious diseases globally (1), underscoring the need to boost innate responses in monocytes and macrophages to enhance early control of *Mycobacterium tuberculosis* (Mtb) infection. Historically, immune responses have been categorized into two arms: innate immunity, which acts rapidly and non-specifically, and adaptive immunity, which is slower but highly specific and long-lasting. However, recent discoveries indicate that innate immune cells can also acquire a nonspecific form of memory, known as trained immunity (2). This process involves recognition of pathogens through pattern recognition receptors, triggering changes in gene expression, metabolism, and chromatin structure that can last for weeks or months after the initial infection. These changes train the cells to respond more effectively to future infections, with the same or unrelated pathogens (3, 4). In the context of TB, such reprogramming may enhance macrophage antimicrobial functions, providing a novel avenue for strengthening early immune defenses against *M. tuberculosis*.

Monocytes and macrophages are highly susceptible to trained immunity after stimulation with *M. bovis* BCG or β-glucan. Following BCG vaccination, human monocytes exhibit a substantial and long-lasting ability to elicit higher responses to secondary stimulation with different pathogens associated with increases in proinflammatory cytokine levels, changes in histone modifications, and gene activation (5). This reprogramming likely occurs at the level of hematopoietic stem cells as β-glucan induces a myeloid differentiation bias, resulting in increased circulating monocytes and their capacity to secrete cytokines in mice (6). Additionally, β-glucan- and BCG-induced trained immunity initiates the functional reprogramming of monocyte metabolism toward aerobic glycolysis, involving AKT activity, mammalian target of rapamycin (mTOR), and hypoxia-inducible factor 1-alpha (HIF-1α), to develop an enhanced response capacity to subsequent stimulation (7, 8).

The increased metabolic activity associated with trained immunity generates a significant number of immunomodulatory metabolites, such as fumarate, succinate, or itaconate, which play a central role in inducing long-term reprogramming of innate immune cells (9). The accumulation of acetyl-CoA induces histone acetylation, fumarate inhibits histone demethylases, thus inducing epigenetic changes in human monocytes, and itaconate inhibits succinate oxidation, causing translocation of the anti-inflammatory transcription factor NRF2 (10–12). Itaconate can induce transcriptional, epigenomic, and metabolic changes characteristic of trained immunity, while producing short-term anti-inflammatory characteristics (13). The metabolic rewiring of innate immune cells regulates their plasticity and epigenetic reprogramming (14). Evidence from human and murine models has demonstrated that trained macrophages exhibit heightened proinflammatory responses, improved pathogen clearance, and epigenetic marks (e.g., H3K27ac, H3K4me3) that prime genes related to immune activation (15–17).

Macrophages are central to controlling *M. tuberculosis* infection by modulating effector mechanisms to limit bacterial replication and expansion, promote the differentiation of antigen-specific T cells, and drive the production of proinflammatory cytokines that promote the migration of other immune cells, with possible involvement of trained immunity (18, 19). This project investigated the potential of harnessing trained immunity to augment macrophage-mediated responses against *M. tuberculosis*. Typical trained immunity inducers, e.g., *M. bovis* BCG, *Candida albicans*, and β-glucan, are still unsuitable for immunotherapy. Because trained immunity is initiated when macrophages recognize conserved pathogen-associated molecular patterns (PAMPs) through their pattern-recognition receptors, we hypothesize that bacterial lysates and whole-bacteria immunomodulators, which contain multiple Gram-negative and Gram-positive microorganisms and are a rich source of PAMPs, such as peptidoglycan, lipoteichoic and teichoic acids, diacyl- and triacyl-lipopeptides, muramyldipeptide, and flagellin (20), may serve as potent inducers of trained immunity in macrophages. Both immunomodulators are already approved for the prevention of recurrent respiratory and urinary tract infections (21–23).

In this study, we first determined that commercially available bacteria-derived immunomodulators induced training-associated transcriptional and phenotypic changes in human macrophages. To assess the influence of macrophage polarization on antimicrobial function, we characterized the trained macrophage phenotype using spectral flow cytometry. Finally, we examined the effect of such training on infection with *M. tuberculosis*. We found that bacteria-derived immunomodulators offer a safe and accessible immunotherapeutic for inducing trained-like signatures in human macrophages.

## 2 Materials and methods

### 2.1 Samples

This study was performed using buffy coats from healthy donors to the blood banks of the Instituto Nacional de Enfermedades Respiratorias Ismael Cosío Villegas (INER) and Instituto Nacional de Ciencias Médicas y Nutrición Salvador Zubirán. Overall, we included 16 healthy subjects. This study was approved by the institutional review boards, which waived informed consent on grounds of donor anonymity and assigned the number C43-22.

### 2.2 Monocyte-derived macrophages

Peripheral blood mononuclear cells were obtained using a Lymphoprep gradient (Axis Shield, San Jose, CA, USA). Monocytes were then isolated by positive selection using CD14+ magnetic bead technology (Miltenyi Biotec, CA, USA) conjugated to anti-CD14 antibodies. Monocytes in suspension were adjusted to 1×10^6^ cells/ml in RPMI-1640 (Lonza, Walkersville, MD), supplemented with 200 nM L-glutamine (Lonza, Walkersville, MD), 5 µg/L gentamicin (Lonza, Walkersville, MD), and 10% human serum (Valley Biomedical, Winchester, VA 22602, USA), herein referred to as complete medium. Human monocytes were cultured for 7 days to generate monocyte-derived macrophages (MDMs).

### 2.3 Bacteria and bacteria-derived immunomodulators

*M. bovis* BCG (ATCC 35743) and *M. tuberculosis* H37Ra (ATCC 25177) were grown at 37°C in 7H9 broth (Difco, Detroit, MI) supplemented with ADC Enrichment (Beckton Dickinson, BD, San Jose, CA), 0.2% glycerol, and 0.05% Tween-80 (Sigma-Aldrich, St. Louis, MO USA), and quantitated by the colony forming units method using solid Middlebrook 7H10 medium (Difco) supplemented with 0.5% glycerol, OADC Enrichment (BD) and 0.05% Tween-80. Aliquots were stored at -80°C until use.

Bacterial lysate Pulmonarom PML (Sanofi-Aventis, México) and whole-bacteria immunotherapeutic IPI Bacterial Suspension (ASAC Pharmaceutical Immunology, ASAC S.A., México) were used in their commercial oral formulations, and the CFU counts were used as reported by the manufacturers in their technical sheets.

### 2.4 Trained immunity model

Monocytes were seeded at 1×10^6^ cells/mL in complete medium, and cultured in the presence of Bacillus Calmette-Guérin (BCG) at an MOI 1, 100 ng/mL lipopolysaccharide (LPS, Sigma-Aldrich, St. Louis, MO USA), bacterial lysate (BL) at 1×10^8^ CFU/ml, and bacterial suspension (BS) at 1×10^6^ CFU/ml, incubated for 24 hours at 37°C in 5% CO_2_. After 24 hours, supernatants were removed and stored at –20°C. The plate was washed twice and replenished with complete medium to differentiate monocyte-derived macrophages (MDMs) for an additional 6 days. On day 6, culture supernatants were collected and stored at –20°C, and the medium was replenished. Secondary stimulation with *M. tuberculosis* H37Ra at an MOI of 5, LPS, or both was carried out for an additional 24 hours, after which the culture supernatants were collected (day 7) and stored at –20 °C until use.

Untrained control macrophages were incubated in complete medium for 6 days and subsequently infected with *M. tuberculosis* H37Ra at an MOI of 5, with LPS, or with both for an additional 24 hours.

In selected experiments, we used 1mM of methyltransferase inhibitor 5'deoxy-5'-(methylthio) adenosine (MTA, brand, country), which can prevent the induction of a trained phenotype (24), for 1 hour before exposing the monocytes to the training stimuli to prevent epigenetic changes. MTA was replenished after the training stimulus was washed. The cells were cultured and infected with *M. tuberculosis* H37Ra as described above.

### 2.5 Cytokine measurements

The levels of pro-inflammatory cytokines IL-12p70, TNF-α, IL-10, IL-6, IL-1β, and IL-8 were measured in the supernatants at 24 hours, day 6, and day 7 using the Cytometric Bead Array (CBA) kit from BD (San José, CA, USA), according to the manufacturer’s instructions. Samples were analyzed using a FACS CANTO II flow cytometer (BD, San José, CA, USA), and results were processed with FCAP software (BD, San José, CA, USA). Preliminary pilot measurements of IL-1β were determined by ELISA following the manufacturer’s instructions (Mabtech, Cincinnati, OH, USA), and TNFα was measured as previously reported (25).

### 2.6 Gene expression

MDMs trained for 7 days as described above were lysed with RLT buffer (Qiagen, Germantown, Maryland, USA) for total RNA extraction using the RNeasy Microkit (Qiagen) according to the manufacturer’s instructions. RNA concentration was subsequently measured using a Nanodrop spectrophotometer (Thermo Fisher Scientific, Millersburg, Pennsylvania, USA), and samples were stored at -80°C until use. Complementary DNA (cDNA) was then synthesized using the SuperScript First-Strand Synthesis System kit (Invitrogen, Millersburg, Pennsylvania, USA), according to the manufacturer’s protocol. Gene expression levels of metabolic enzymes PFKP, HK2, GLUD1, and GLS, as well as genes associated with antimicrobial activity such as CAMP, NPC2, MAP1LC3, and ATG16L1, were analyzed by real-time PCR using TaqMan technology and a QuantStudio 12 Flex system (Applied Biosystems, Waltham, USA). Expression differences were calculated using the 2^−ΔΔCt method, with B2M as the housekeeping gene. Details of the TaqMan assays appear in **Supplementary Table 1**.

### 2.7 Macrophage phenotype

Trained MDMs were detached using ice-cold PBS (Corning™Mediatech™ Glendale, Arizona, USA), transferred to polypropylene tubes, washed twice with cold PBS, and centrifuged at 1500 rpm for 5 minutes. The supernatant was discarded, and 1 µL of viability dye AquaDye (Invitrogen) was added and incubated at room temperature in the dark for 20 minutes. After incubation, cells were washed, centrifuged, and the supernatant was discarded. The cell pellet was then resuspended in 2 µL of SBF (Invitrogen) plus 50 µL of Brilliant Stain Buffer, followed by the addition of the antibody cocktail: CXCR4-PE-Cy5 (Biolegend, San Diego, CA, USA) CD3-BV510, CD14 PE-CF594, CD16-APC-H7, CD163-BUV395, TLR2-BUV615, TLR4-PE, TLR9-APC, CD64-PE-Cy7, HLA-DR R718, CD36-BV605, CD80-BV421, CD86-BV650, CCR2-BV711, CD209-FITC, and CD206-BV786 (Becton Dickinson, BD, San José, CA, USA), additional details in **Supplementary Table 1**). Cells were incubated for 30 minutes at 4°C in the dark. After staining, cells were washed twice with cold PBS and resuspended in a final volume of 200 µL PBS.

Single-stained controls for spectral unmixing were prepared using BD CompBeads Plus (BD) following the manufacturer’s instructions. All samples were acquired using the FACS Discover S8 spectral flow cytometer with integrated CellView and FACS Chorus software (BD). Spectral unmixing was carried out using the single-stained controls and unstained samples, allowing for autofluorescence subtraction and deconvolution of overlapping emission spectra. Data analysis was performed using FlowJo v10.10 (BD). Dimensionality reduction analysis was conducted using t-distributed stochastic neighbor embedding (t-SNE) and FlowSOM for unsupervised quantitative clustering.

### 2.8 Statistical analyses

The macrophage’s phenotype was only described, not compared. Multiple comparisons were performed using one-way ANOVA followed by Dunnett’s *post hoc* test. Relative gene expression was assessed with a one-sample Wilcoxon test. Analyses were conducted using GraphPad Prism 8.0, considering p-values < 0.05 statistically significant.

## 3 Results

### 3.1 Immunomodulatory properties of BS and BL

Given that BS and BL were liquid pharmaceutical formulations sold for oral administration, we first evaluated their ability to stimulate human monocytes *in vitro*. A pilot study was conducted to identify concentrations that elicited immunostimulatory activity without cytotoxicity (**Supplementary Figure 1**). To further characterize their immunomodulatory potential, we quantified the production of IL-12p70, TNFα, IL-10, IL-6, IL-8, and IL-1β in monocytes stimulated for 24 hours with BS (1 × 10^6^ CFU/mL) or BL (1 × 10^8^ CFU/mL). Lipopolysaccharide (LPS) and *M. bovis* BCG were included as positive controls, while unstimulated cells cultured in medium served as the negative control.

**Figure 1:**
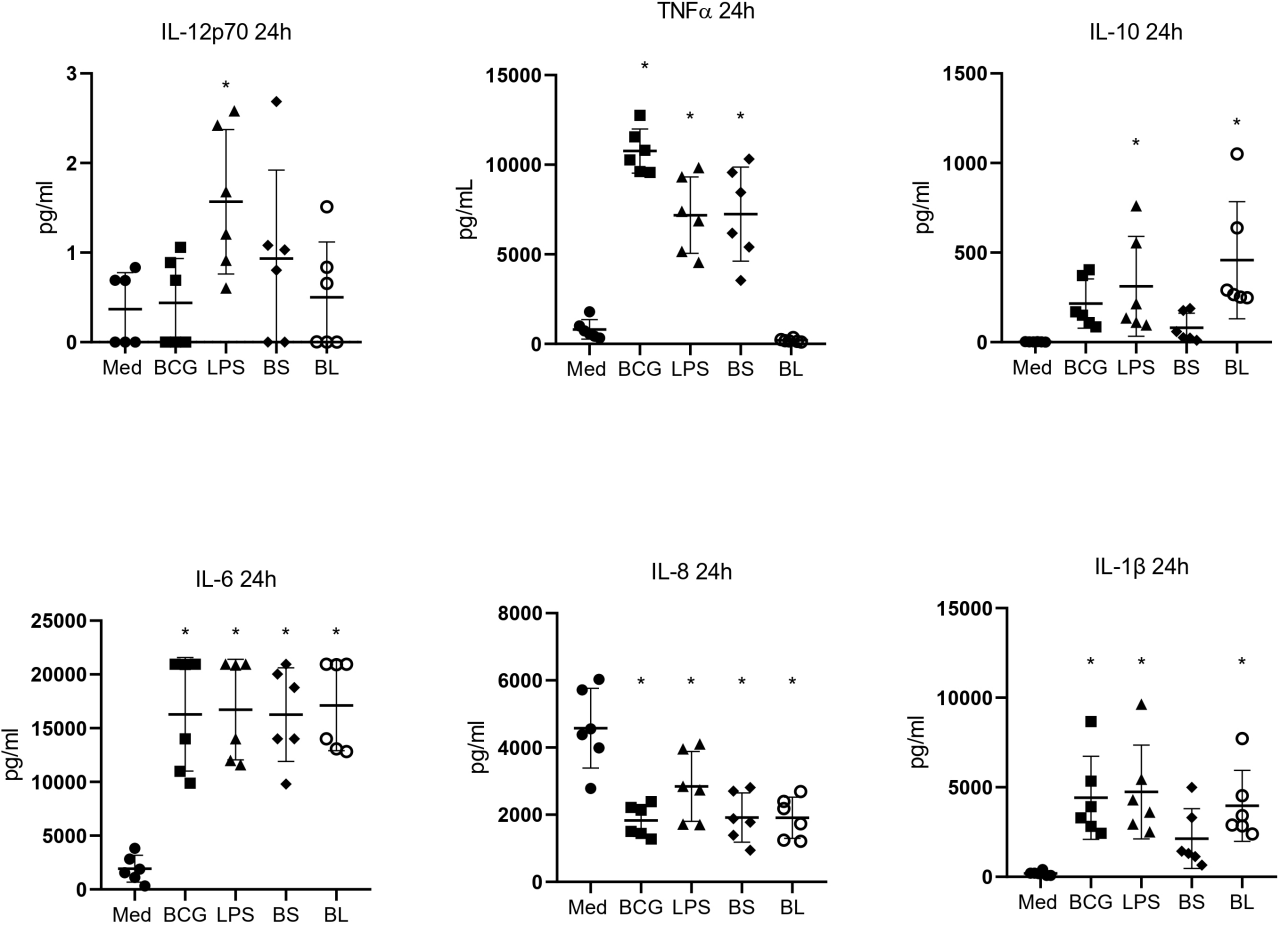
BS and BL formulations induced innate responses in human monocytes. Monocytes were cultured in the presence of BS (1x10^6^ UFC/ml) and BL (1x10^8^ UFC/ml) for 24 hours. We included 100 ng/mL of LPS and infection with BCG at an MOI of 1 as a positive control, and medium as a negative control. Cytokines IL-12p70, TNFα, IL-10, IL-6, IL-8, and IL-1β were measured in the supernatants by flow cytometry using a CBA assay (n=6). Means ± SD are depicted. *p<0.05 compared to Medium. One-way ANOVA, followed by Dunnett *post hoc* for multiple comparisons.

BS stimulation significantly increased the monocytes’ production of TNF-α and IL-6. In contrast, BL stimulation induced significant levels of IL-6, IL-1β, and IL-10, suggesting the activation of distinct immunological pathways. Surprisingly, all stimulators reduced the expression of IL-8. As expected, LPS elicited robust production of all measured cytokines, whereas BCG infection selectively induced proinflammatory cytokines (**Figure 1**).

### 3.2 BS and BL induce trained immunity-like signatures in macrophages

**Figure 2:**
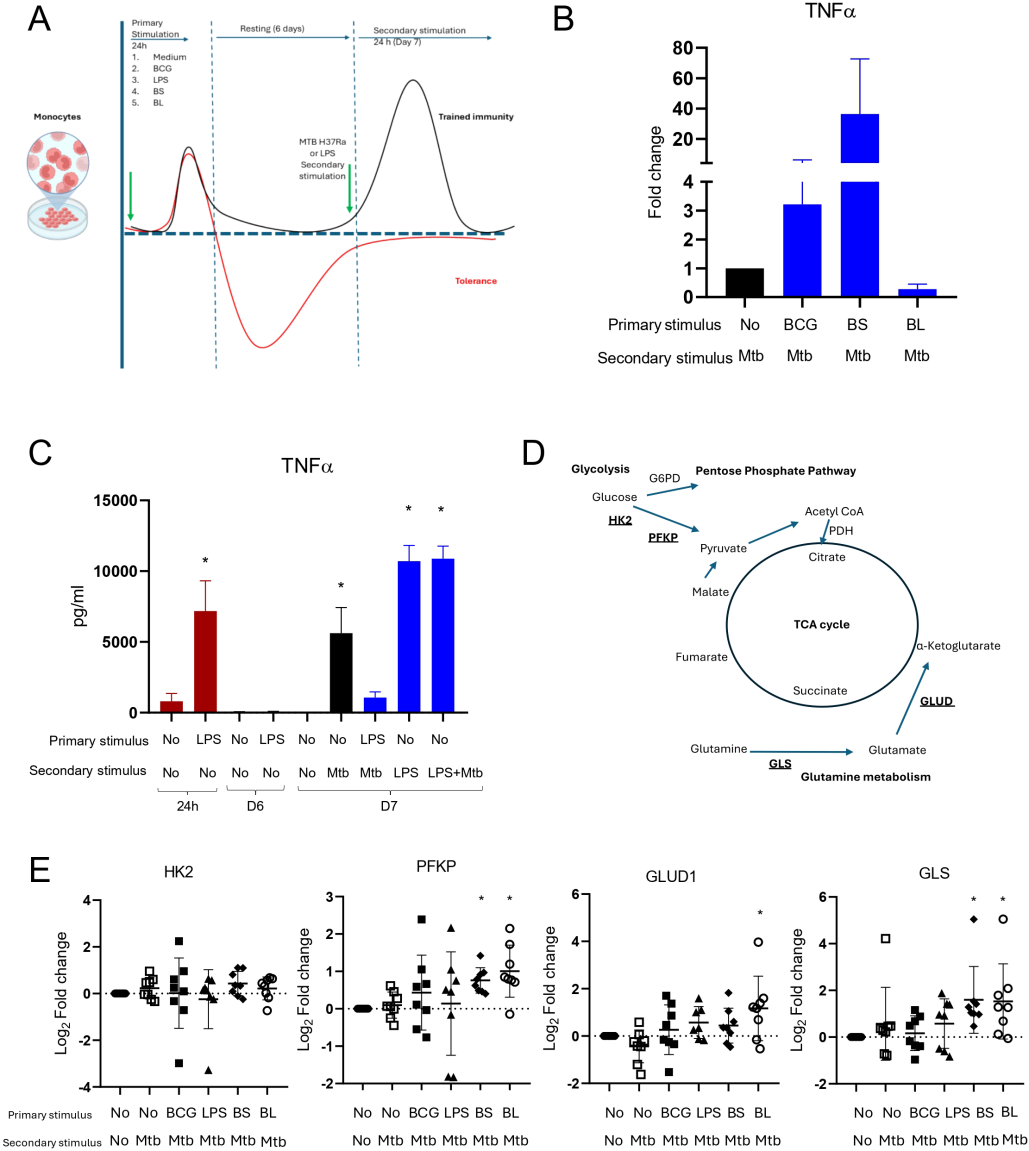
Induction of trained immunity. **(A)** Schematic representation of the training protocol in primary human monocytes. Cells were stimulated with a primary stimulus for 24 hours. The first stimulus was washed out, followed by a 6-day resting period. On day 6, the secondary stimulus was incorporated, and cells were cultured for an additional 24 hours. Supernatants were collected at 24h, day 6, and 24h post-infection (day 7). Primary stimulus included medium, BCG (MOI 1), LPS (100ng/ml), bacterial suspension (BS, 1x10^6^ UFC/ml), and bacterial lysates (BL, 1x10^8^ UFC/ml). The secondary stimuli were LPS (100ng/ml) and *M. tuberculosis* H37Ra (MOI 5). **(B)** Untrained macrophages were infected with *M. tuberculosis* H37Ra (MOI 5), and their TNF-α production was set at 1. Responses of trained macrophages are reported as the fold change in TNF-α production relative to untrained macrophages. Depicted are the Means ± SEM (n=6). **(C)** Monocytes stimulated with LPS following the training protocol. TNF-α production was measured at 24 hours, on day 6 (D6), and after *M. tuberculosis* infection on day 7 (D7). Means ± SD are depicted; *p<0.05, one-way ANOVA followed by Dunnett’s *post hoc* (n=6). **(D)** Schematic of the tricarboxylic acid cycle (TCA) depicting the intervention points for the enzymes associated with trained immunity, glycolysis (HK2 and PFKP), and glutamine (GLS and GLUD) metabolism. **(E)** Trained macrophages infected with *M. tuberculosis* for 24 h were lysed and retrotranscribed for gene expression assessment by qPCR. Log_2_ Fold changes relative to untrained macrophages were calculated. Depicted are individual results with means and SD; *p<0.05, one-way ANOVA followed by Dunnett’s *post hoc* (n=8).

Next, we investigated whether BS and BL induce trained immunity in monocytes, enhancing responses to *M. tuberculosis* infection. We used a training protocol previously reported for BCG training (24), which involved applying a primary stimulus to monocytes, followed by a washout and a 6-day resting phase (**Figure 2A**). On day 6, the cells, now differentiated into MDMs, received a secondary stimulus, followed by a 24-hour incubation period. TNF-α production of trained MDMs was measured at day 7 and reported as fold change relative to untrained MDMs’ production (**Figure 2B**). Untrained MDMs infected with *M. tuberculosis* were used as the reference henceforth. MDMs trained with BCG exhibited enhanced TNF-α production in response to *M. tuberculosis* infection, consistent with trained-like signatures. MDMs trained with BS or BCG and infected with *M. tuberculosis* had the highest response, although it did not reach significance. Training with BL did not affect the TNF-α response (**Figure 2B**).

Because we did not know whether the large amounts of PAMPs present in BS and BL, many of which originated from gram-negative bacteria, would result in tolerance rather than activation, we included lipopolysaccharide (LPS), a known inducer of tolerance, for comparative purposes. At 24 hours, LPS induced robust TNF-α production, which was lost after the 6-day rest. The LPS-prestimulated MDMs were unresponsive to a secondary stimulation with *M. tuberculosis*, consistent with tolerance. Regular MDMs produced the expected levels of TNFα when stimulated with LPS for 24 h or infected with *M. tuberculosis* and then stimulated with LPS. This indicates that LPS produced tolerized macrophages during the 6-day training protocol rather than inducing trained immunity (**Figure 2C**, **Supplementary Figure 2**).

To confirm trained immunity and its metabolic regulation, we assessed expression of enzymes involved in glycolysis (Hexokinase 2, HK2, and Phosphofructokinase, PFKP) and glutamine metabolism (glutaminase, GLS, and glutamate dehydrogenase 1, GLUD1) (**Figure 2D**). Significant increases in PFKP and GLS gene expression were observed with BS and BL treatment compared to untrained MDMs. BL also significantly upregulated GLUD1. No significant differences in HK2 expression were observed. These findings suggest that BS and BL likely induce trained immunity, primarily by enhancing PFKP and glutamine metabolism. BCG tended to increase expression of all four genes, but this did not reach significance; however, BS and BL responses were higher than those during training with BCG, suggesting their role in the metabolic reprogramming underlying training-associated transcriptional and phenotypic changes (**Figure 2E**).

### 3.3 Characterization of trained macrophage phenotype

The morphological and functional diversity of macrophages plays a key role in either controlling or facilitating the dissemination of *M. tuberculosis*. In simplified terms, M1 macrophages are pro-inflammatory and can eliminate or contain intracellular pathogens, whereas M2 macrophages tend to support intracellular persistence and are often associated with tissue repair and immunoregulation (26). Recent evidence suggests that the phenotype of trained macrophages is strongly influenced by the nature of the training stimulus (27, 28). To explore this, we used spectral flow cytometry combined with dimensionality reduction techniques to characterize macrophage phenotypes in our model.

**Figure 3:**
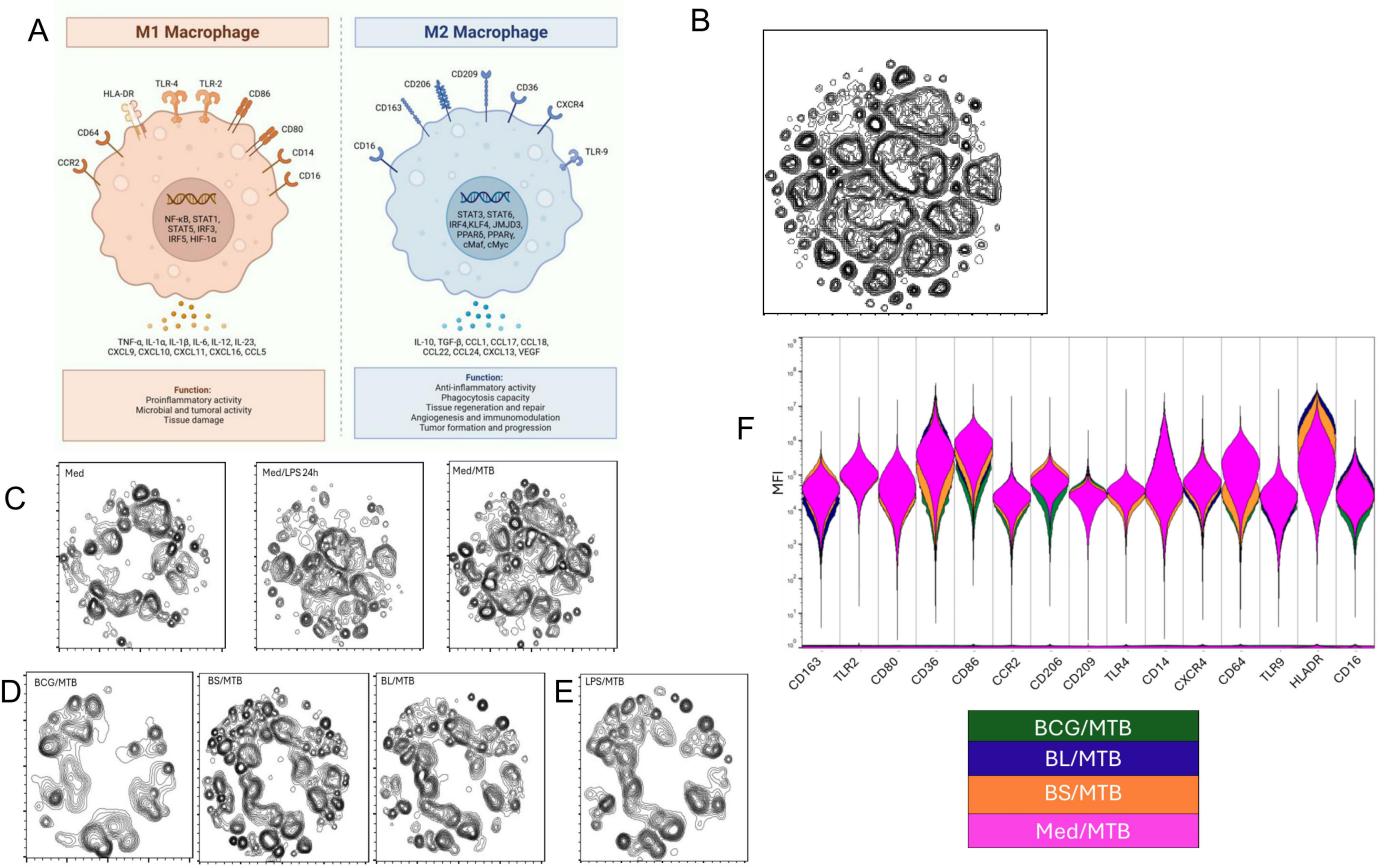
Immunophenotype of trained macrophages. **(A)** Schematics of M1 and M2, typical membrane markers, and functional phenotypes. **(B)** Macrophages were infected with *M. tuberculosis* (MOI 5) for 24 (h) Cells were stained with antibodies anti CD80, CD86, TLR2, TLR4, HLA-DR, CD64, CCR2, CD14, CD16, CD206, CD209, CD163, CD36, CXCR4, and TLR9 to determine their expression by spectral flow cytometry. All live single macrophages from 6 individuals were concatenated (2,405,332 total events) and analyzed using dimensionality reduction via t-SNE. The untrained **(C)**, trained **(D)**, and tolerized **(E)** macrophage phenotype profile is depicted (See the gating strategy in **Supplementary Figure 3** and individual markers expression in **Supplementary Figure 4**) **(F)** Spectral population viewer of trained macrophages in comparison with untrained macrophages. Violin plots depict the range of expressions for each marker.

We defined M1 macrophages by surface expression of CD80, CD86, TLR2, TLR4, HLA-DR, CD64, CCR2, CD14, and CD16, and M2 macrophages by expression of CD206, CD209, CD163, CD36, CXCR4, TLR9, and CD16 (**Figure 3A**). Cells from trained and untrained macrophages infected with *M. tuberculosis* were collected from six individuals and concatenated for analysis (see gating strategy in **Supplementary Figure 3**). We then performed t-SNE on day 7, 24 h post-infection, to visualize the patterns of the 15 surface markers’ expression (**Figure 3B**). The individual expression profiles for each marker are shown in **Supplementary Figure 4**.

The t-SNE analysis revealed distinct clustering profiles among untrained MDMs depending on the condition: uninfected, stimulated with LPS, or infected with *M. tuberculosis* (**Figure 3C**). In contrast, trained MDMs, whether primed with BCG, BS, or BL, displayed more homogeneous clustering patterns after *M. tuberculosis* infection, suggesting convergence toward a typical trained phenotype, although each condition retained unique characteristics (**Figure 3D**). Interestingly, macrophages tolerized with LPS showed an expression pattern that partially overlapped with that of BL-trained MDMs, suggesting that differences between immune tolerance and certain trained states may be in the functionality of signaling pathways (**Figure 3E**). The violins in the spectral viewer showed that the relative expression of the markers of interest differ between trained and untrained MDMs infected with *M. tuberculosis*, suggesting that the differences in the expression profile may be attributed to the training (**Figure 3F**). For instance, in untrained MDMs, *M. tuberculosis* infection shifted the MDMs general phenotype toward a mixed phenotype with M1 (CD86^hi^HLA-DR^hi^CD14^hi^CD64^hi^CXCR4^hi^) and M2 (CD163^hi^CD36^hi^) markers highly expressed, consistent with a flexible state. However, BCG-, BS-, and BL-trained MDMs exhibited the highest expression of M1 markers.

### 3.4 Macrophages trained with BCG, BS, and BL exhibit an M1 phenotype

**Figure 4:**
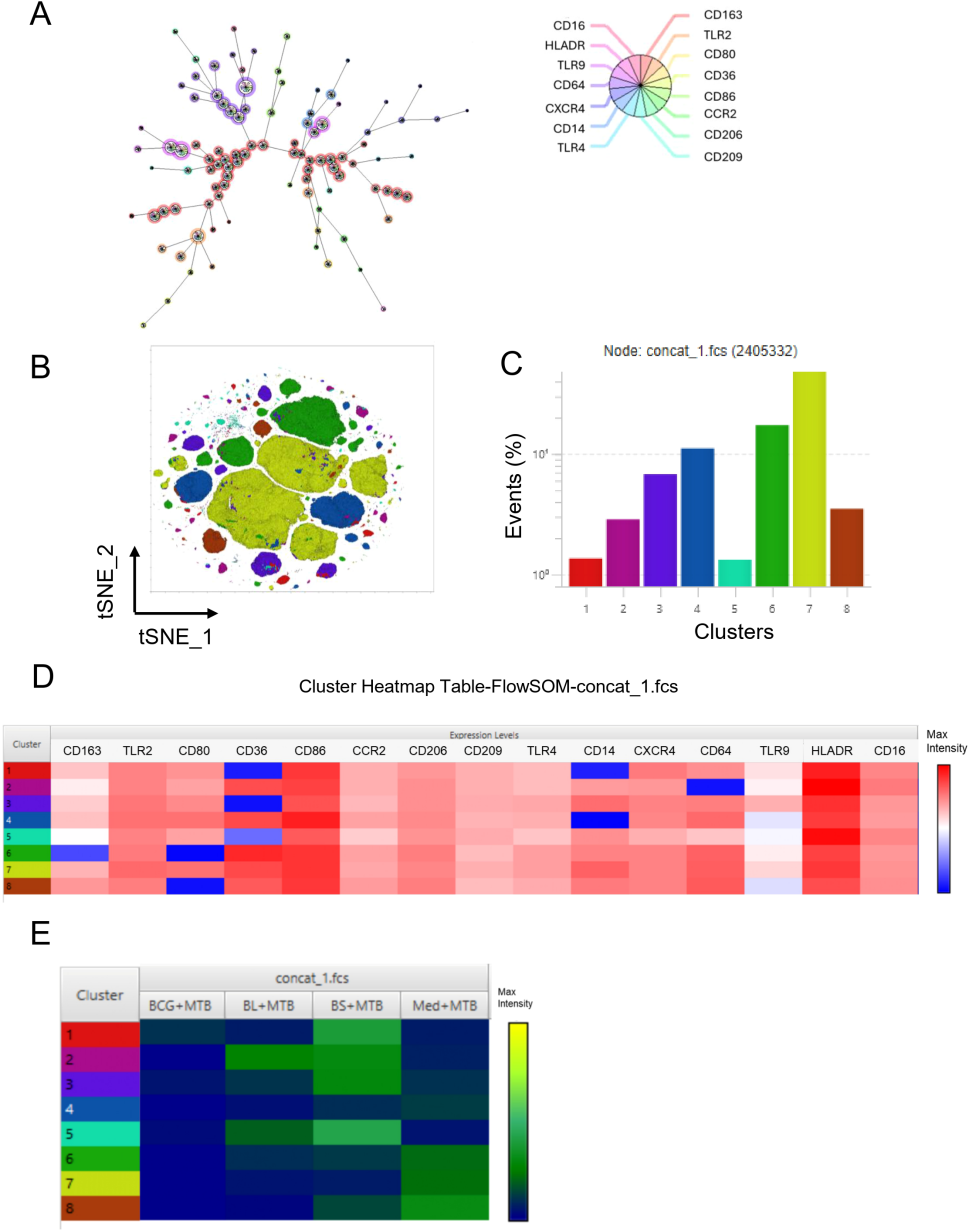
Cluster analysis of the trained macrophages’ phenotype. After dimensionality reduction (n=6), clusters of macrophage subpopulations were determined using FlowSOM. **(A)** The FlowSOM node map shows a marker-based clustering structure. Eight metaclusters were identified; **(B)** t-SNE projection of metaclusters illustrating phenotypic distribution across conditions. Clusters were further compared between experimental conditions using ClusterExplorer. **(C)** Frequency of each metacluster. **(D)** Heatmap showing relative expression of 15 surface markers across metaclusters with blue-red expression level range. The color bar, with maximum intensity in red, indicates the expression level, and the absence of expression is represented in blue. **(E)** Metacluster distribution across experimental conditions. The Cluster Heatmap shows, in blue to yellow, the frequency of the clusters, with yellow at the maximum intensity (most abundant).

Because MDMs are expected to be a mixture of phenotypically different subpopulations, we performed unsupervised clustering using FlowSOM, followed by metacluster annotation with Cluster Explorer to further characterize phenotypic diversity among macrophages (**Figures 4A, B**). We identified eight distinct macrophage clusters across all experimental conditions using expression data from 15 surface markers associated with M1 and M2 polarization (**Figure 4C**). **Figure 4D** shows the expression level for each marker in each cluster. In general, TLR9 was the lowest expressed marker, followed by CD163. CD86 and HLA-DR were highly expressed, whereas CD14, CD36, CD64, CD80, CCR2, and CXCR4 were differentially expressed. The most abundant macrophage subpopulation (cluster #7, **Figure 4C**) exhibited high levels of CD86, CD64, and HLA-DR, as well as medium levels of CD80, CD36, CD14, and TLR2, all of which indicate an M1 phenotype.

Next, we investigated which macrophage subpopulation was present across experimental conditions (**Figure 4E**). The color bar in the heatmap indicates the frequency of each cluster across conditions. Untrained MDMs infected with *M. tuberculosis* (Med+Mtb) had a higher abundance of clusters 6, 7, and 8, which exhibit a mixed phenotype with high expression of both M1 and M2 markers. Those clusters were less abundant in trained MDMs. BS-trained macrophages showed selective enrichment in M1 phenotype clusters with high expression of CD80, CD86, HLA-DR, TLR2, and CD16, and low expression of CD163, TLR9, CCR2, and TLR4 (clusters 1, 2, 3, and 5). In fact, BS-trained macrophages exhibited a greater diversity of macrophage subpopulations. BL-trained macrophages were mainly enriched in clusters 2 and 5.

**Figure 5:**
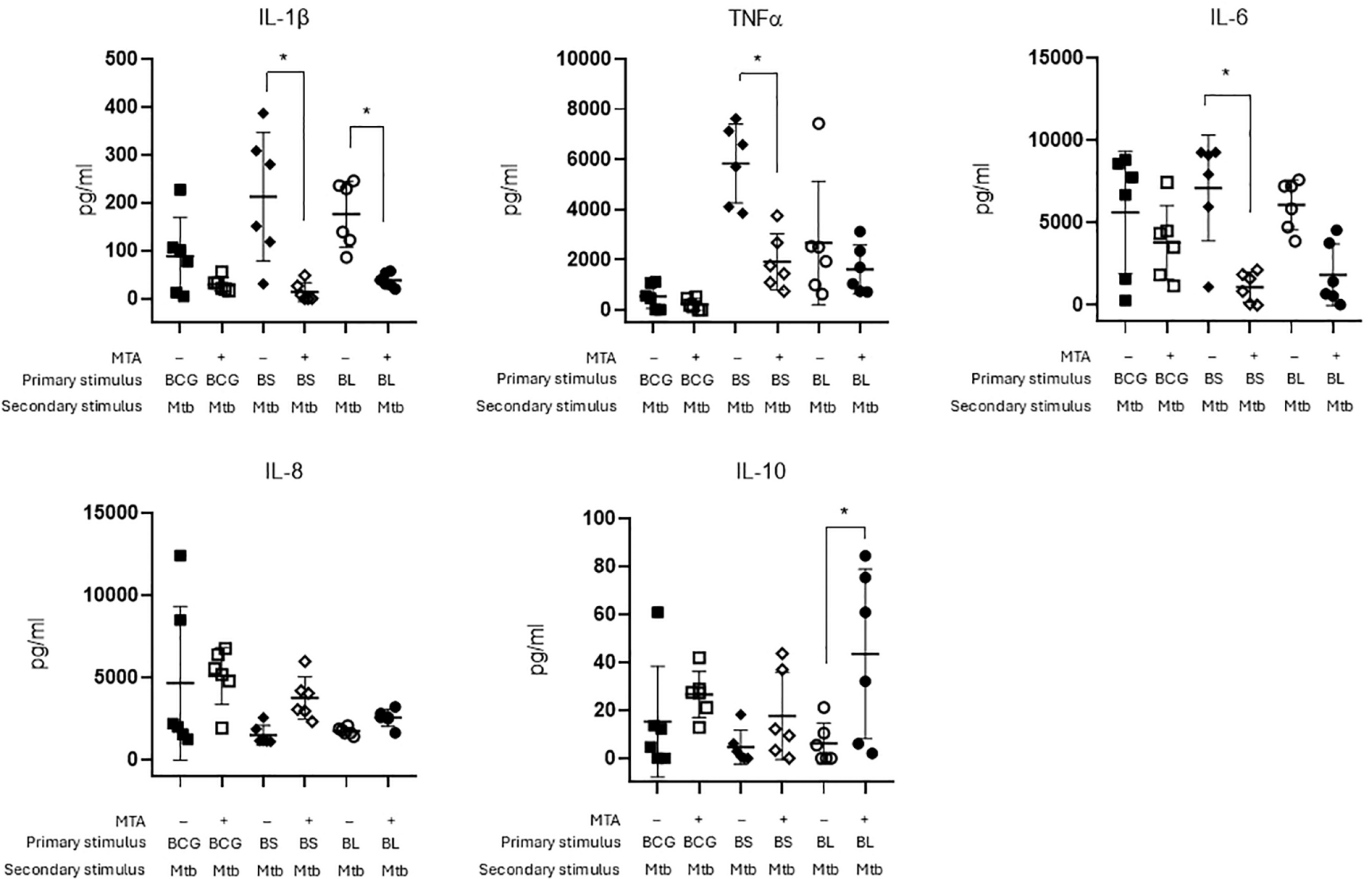
Cytokine production of trained macrophages. Monocytes were trained with BCG, BS, and BL in the presence or absence of 1mM of 5'-deoxy-5'-(methylthio) adenosine (MTA) for 24 h. The training stimuli were washed, and the macrophages were allowed to differentiate for 6 days. Trained macrophages were infected with *M. tuberculosis* for 24 h, and cytokine production was measured in the supernatants by flow cytometry using CBA technology. Depicted are individual results with means and SD. *p<0.05 compared with untrained macrophages infected with Mtb. One-way ANOVA followed by Tukey’s *post hoc* (n=6).

To further characterize the trained macrophages’ phenotype, we evaluated the production of IL-1β, TNF-α, IL-6, IL-12p70, IL-8, and IL-10 cytokines in the culture supernatants 24 h after *M. tuberculosis* infection. Here, macrophages were incubated in the absence or presence of MTA, an inhibitor of histone 3 lysine 4 trimethylation (24), thereby inhibiting epigenetic reprogramming before the training stimulus was administered. BS- and BL-trained MDMs produced higher IL-1β and TNF-α than BCG-trained macrophages, supporting their capacity to induce a trained proinflammatory state, consistent with the M1 phenotype. This phenotype was significantly inhibited by preincubation with MTA before training (**Figure 5**), highlighting the dependence of epigenetic reprogramming for training. Interestingly, training did not affect IL-6, IL-8, and IL-10 production, suggesting other responses associated with *M. tuberculosis* infection were not triggered. Levels of IL-12p70 were negligible, likely due to the absence of lymphocytes in the culture (data not shown).

### 3.5 Trained macrophages express enhanced antimicrobial mechanisms

**Figure 6:**
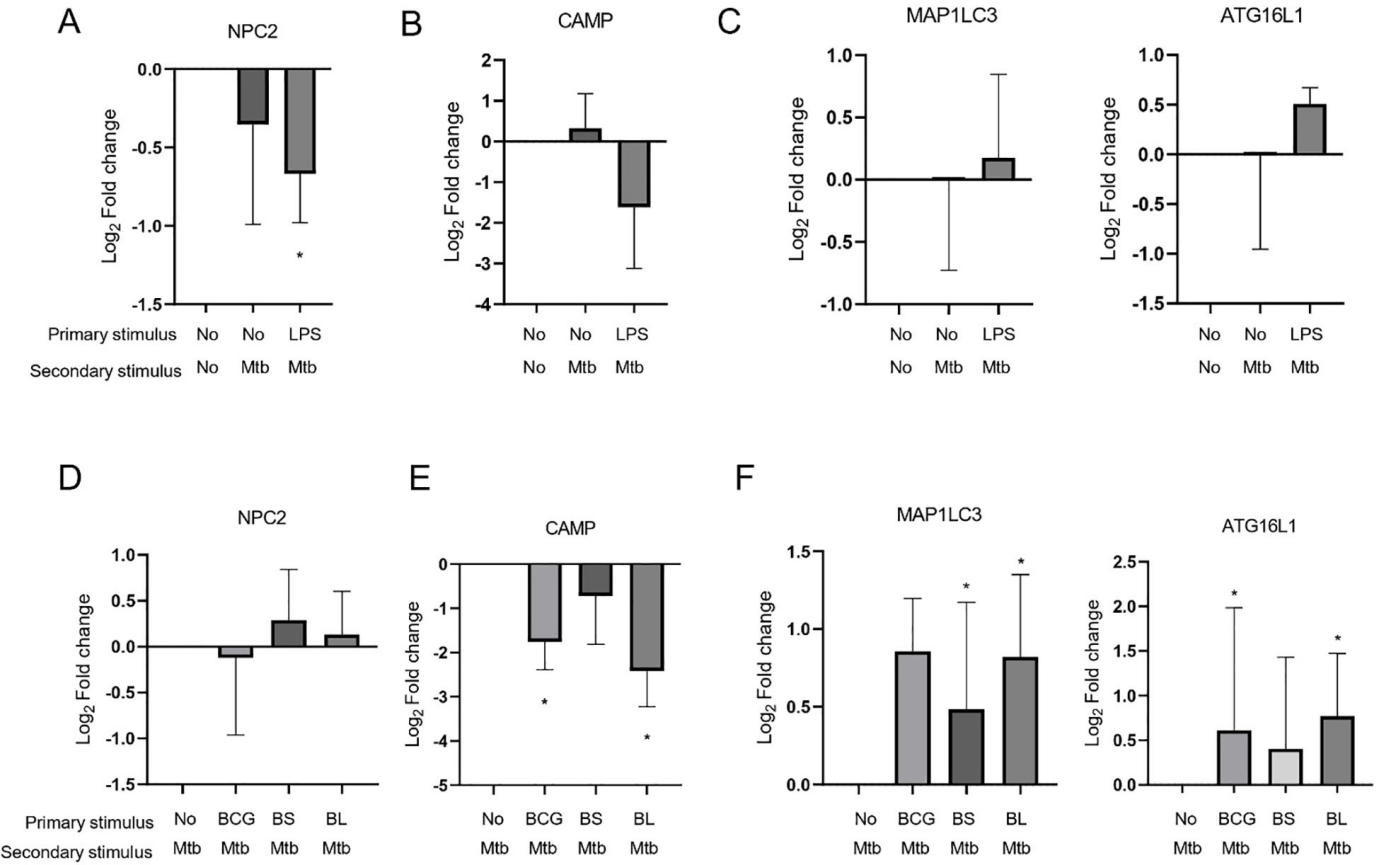
Gene expression of antimicrobial effectors of trained macrophages. Untrained macrophages were infected with *M. tuberculosis* for 24 h, lysed, and retrotranscribed to determine the gene expression of NPC2 **(A)**, CAMP **(B)**, and autophagy-related MAP1LC3 and ATG16L1 **(C)** by qPCR. Log_2_ Fold changes relative to uninfected macrophages were calculated. Depicted are medians and IQR; *p<0.05, Wilcoxon’s one-sample test (n=8). Gene expression of NPC2 **(D)**, CAMP **(E)**, and MAP1LC3 and ATG16L1 **(F)** of trained macrophages infected with *M. tuberculosis* was measured. Log_2_ Fold changes relative to untrained macrophages were calculated. Depicted are medians and IQR; *p<0.05, Wilcoxon’s one-sample test (n=8).

Because M1 macrophages possess antimicrobial properties, we measured the expression of genes involved in macrophage antimicrobial responses (NPC2, CAMP, MAP1LC3, and ATG16L1) in trained MDMs. In untrained or LPS-tolerized MDMs, the infection alone downregulated the expression of all four genes relative to uninfected MDMs (**Figures 6A–C**). We determined gene expression relative to uninfected, untrained MDMs to assess the effect of *M. tuberculosis* infection in trained MDMs. BS and BL training restored macrophage ability to upregulate NPC2 and the autophagy-related genes MAP1LC3 and ATG16L1 upon Mtb infection (**Figures 6D, F**). BCG-trained MDMs only upregulated the expression of the autophagy-related genes upon *M. tuberculosis* infection (**Figure 6F**). CAMP expression was downregulated upon *M. tuberculosis* infection, regardless of the immunomodulation status of the MDMs.

## 4 Discussion

BCG-induced trained immunity, a memory-like response in innate immune cells that enhances protection against various pathogens beyond *Mycobacterium tuberculosis*, is characterized by epigenetic and metabolic reprogramming of monocytes and macrophages, leading to increased cytokine production upon secondary stimulation (29–31). In addition to the BCG vaccine, other trained immunity inducers, such as β-glucan, have proven helpful for *M. tuberculosis* control, but are not ready for human therapy (32, 33). This study explored the potential of inducing trained immunity-associated signatures in macrophages as a novel therapeutic strategy to improve control of TB infection using bacteria-derived immunomodulators. Orally administered bacterial suspensions (BS) and lysates (BL) are immunotherapeutics approved for the prevention of recurrent respiratory tract infections and asthma and allergy episodes in children and older adults (34–36). However, their capacity to induce trained immunity and macrophage reprogramming relevant to *M. tuberculosis* infection remains underexplored. Here, we stimulated human macrophages with pharmaceutical preparations of bacterial suspension (BS) and bacterial lysates (BL). We assessed their ability to induce trained immunity and modulate macrophage responses to *M. tuberculosis* infection.

Because we used pharmaceutical oral formulations, we first investigated whether BS and BL were suitable for *in vitro* research. We found they were indeed immunostimulatory, because they differentially induced TNF-α, IL-1β, IL-6, and IL-10 production and had no toxic effect on cell viability. Specifically, BL induced IL-6 and IL-1β production, whereas BS augmented TNF-α and IL-6 levels. Interestingly, BL also increased IL-10 levels, consistent with IL-10 being overexpressed during *S. aureus* infection to prevent bacterial dissemination (35). Surprisingly, IL-8 was downregulated by all immunomodulators. Although there is evidence that some bacteria, such as *S. aureus*, can inhibit IL-8 production, thereby increasing susceptibility to infection (36), activating innate immunity without increasing neutrophil influx by these immunomodulators would indeed be a benefit worth further investigation. Further, we determined the ability of BS and BL to induce trained immunity. We observed the typical signatures of trained immunity, such as enhanced TNF-α production upon a secondary stimulus applied 6 days after priming, and upregulation of glycolysis and glutamine metabolism enzymes. Primary human monocytes trained with β-glucan, *Candida albicans*, and BCG rapidly shift monocyte metabolism toward glycolysis and upregulate genes involved in lipid metabolism and biosynthesis (37). Cytokines TNF-α, IL-1β, and IL-6 are hallmarks of trained immunity. Thus, BS and BL can induce a trained-like immunity in human MDMs. The differences in cytokine production patterns between BS and BL may mainly reflect the fact that BS contains whole bacteria, whereas BL contains lysed bacteria.

Specifically, we observed that BS and BL upregulated the expression of enzymes relevant to glycolysis and glutamine metabolism. Glutamine is a primary energy substrate for lymphocytes and macrophages, as well as a precursor to nucleotides. It is necessary for phagocytic activity (38). Glutamine and glucose are poorly oxidized by macrophages, but they may produce important precursors for DNA, RNA, protein, and lipid synthesis. Moreover, other effects of glutamine metabolism may influence cellular activation during trained immunity. Glutamate can be utilized for ATP and NADPH production, as well as for the production of acetyl-CoA, which serves as a substrate for acetyltransferase enzymes. Notably, H3K27Ac is an important histone mark associated with active promoters in trained monocytes (39).

Macrophage polarization influences antimicrobial function. Macrophages can adopt different activation states or phenotypes, classically activated (M1) and alternatively activated (M2), which have distinct functional profiles. M1 macrophages demonstrate a host‐protective profile during early *M. tuberculosis* infection, with enhanced autophagy marked by increased MAP1LC3 lipidation, ATG expression, and efficient lysosomal trafficking, heightened antigen presentation, and a pro‐inflammatory transcriptional program producing high levels of IL‐12, TNF‐α, IL‐1β, and nitric oxide that restrict bacterial growth (40, 41). In contrast, M2 macrophages consistently exhibit reduced autophagy and antigen presentation, along with increased levels of IL‐10, IL‐4, and specific chemokines (e.g., CCL18, CCL22), thereby establishing an anti-inflammatory milieu that permits bacterial persistence (42).

When we investigated the phenotype of BS- and BL-trained MDMs, we observed a heterogeneous mixture of macrophage subpopulations in the culture. Still, we found that training elicited the generation of unique subpopulations compatible with an M1 phenotype, with high expression of HLA-DR, CD80, CD86, highly relevant for antigen presentation, and TLR4 for innate responses readiness.

These phenotypic differences have functional implications. The subpopulations enriched in trained macrophages likely reflect a shared reprogramming toward enhanced responsiveness and activation thresholds. This may prime them for more effective early containment of intracellular pathogens such as *M. tuberculosis*. In contrast, the mixed clustering of untrained macrophages highlights their limited and context-dependent responses to infection. The resemblance between LPS tolerance and BL training could point to alternative pathways of immune adaptation, potentially balancing pro-inflammatory and regulatory functions during chronic or repeated microbial exposure. These findings suggest that training with BS and BL reshape macrophage phenotypes and influence the host response to *M. tuberculosis*. This offers insight into how microbial-derived agents might be leveraged to improve innate immune preparedness.

The macrophage phenotype is relevant and central to the mechanisms and therapeutic potential of trained immunity in TB. The success of trained immunity-based interventions likely depends on the induction of macrophage states that are functionally optimized for early and effective control of *M. tuberculosis*. For instance, BCG induces a pro-inflammatory, bactericidal type 1 (M1) polarization phenotype in infected macrophages, involving the thrombospondin-TLR pathway (43). Trained immunity induced by damage-associated molecular patterns (DAMPs) produces anti-inflammatory macrophages (44). Metabolic reprogramming may generate atypical pro-inflammatory M2 macrophages (45). Here, we found that BS and BL generated a more diverse and heterogeneous macrophage population than BCG, which shifted towards the antimicrobial M1 phenotype, whereas untrained macrophages comprised mixed M1 and M2 populations. Moreover, cytokine production was consistent with the M1 phenotype and was dependent on epigenetic reprogramming, because it was reversed when MTA, an H3K4me3 inhibitor, was used before training; at a minimum, our observations were partially attributable to histone methylation.

To further confirm the M1 phenotype, we determined gene expression of antimicrobial effectors. We selected NPC2, critical for macrophage antimicrobial activity against *M. tuberculosis* by regulating cholesterol levels and lysosomal acidification (46), cathelicidin antimicrobial peptide (CAMP), also known as LL-37, and autophagy-related MAP1LC3 and ATG16L1, because their expression has been previously reported to participate in macrophage antimicrobial activity against *M. tuberculosis*, especially in the context of immunomodulation with bacteria-derived molecules (47–50). In untrained MDMs, the infection with *M. tuberculosis* downregulated the expression of all four genes, likely related to immune evasion. MDMs trained with BS and BL restored their ability to upregulate NPC2, MAP1LC3, and ATG16L1 after infection with *M. tuberculosis*. Unexpectedly, CAMP remained downregulated even in trained macrophages, which suggests selective activation of antimicrobial programs associated with trained immunity. In this regard, CAMP expression is not consistently induced in human macrophages by many common bacterial stimuli, including Salmonella (51) and *Mycobacterium tuberculosis* (49), and its induction may require a different type of inducer, such as vitamin D. Further evaluations on bacterial killing will define the full extent of the antimicrobial programs activated in BS- and BL-trained macrophages.

BS and BL induced trained immunity-like features that influenced their responses to *M. tuberculosis* infection in human MDMs, with distinct differences between them and BCG. Compared with BCG, both BS and BL increased the expression of genes involved in glucose and glutamate metabolism, NPC2, and autophagy. BCG broadly altered macrophage phenotype by downregulating more surface markers, whereas BS and BL favored M1-like subsets, with BS also showing greater phenotypic diversity. Cytokine profiles diverged: BS responses resembled those of BCG, while BL induced higher IL-1β expression. These differences likely reflect the nature of the stimuli: BCG as a single microorganism, BS as a bacterial mixture, and BL as a mixture of PAMPs.

The most striking observation was the absence of TNF-α enhancement with any training stimulus. While this regulated response may be clinically beneficial, it contrasts with prior studies. BCG vaccination enhances trained immunity and boosts innate and adaptive responses to *M. tuberculosis* (52, 53). Similarly, BCG training enhances TNF-α and IL-6 production in response to LPS stimulation (54), whereas β-glucan, a single PAMP, promotes IL-1β–dependent trained immunity against *M. tuberculosis* (32). In the setting of TB, a macrophage phenotype that favors IL-1β production, upregulation of antimicrobial effectors, and antigen presentation is more likely to control bacterial replication. Therefore, the ability to influence or stabilize a macrophage phenotype with enhanced antimicrobial activity is central to the rationale for trained immunity-based interventions. Identification of well-defined compounds that induce trained immunity and are already approved for human therapy can improve our understanding of innate immune memory and broaden the scope of its clinical applications.

This study has limitations. The study focuses primarily on indicative signatures and therefore does not yet encompass all dimensions of microbial control, cellular reprogramming, or long-term immune modulation within the framework of trained immunity. While our results highlight relevant trends, broader mechanistic and comparative approaches remain to be explored. These aspects offer opportunities for future refinement and expansion, thereby strengthening and contextualizing the emerging insights presented here.

## 5 Conclusions

Our results demonstrate that oral formulations of bacterial suspension and bacterial lysates can induce training-associated transcriptional and phenotypic changes in human macrophages, including increased cytokine responses, phenotypic activation, and transcriptional reprogramming. Given the central role of trained macrophages in the early containment of *Mycobacterium tuberculosis*, these immunological signatures point towards new adjunctive strategies to boost innate responses in tuberculosis and other chronic infections. Further *in vivo* validation is warranted to define their therapeutic potential.

## Data Availability

The original contributions presented in the study are included in the article/**Supplementary Material**. Further inquiries can be directed to the corresponding author.
