# Supplementary material for: Immunomodulation by bacterial products promotes innate signatures favorable to macrophage responses in tuberculosis infection
Source: Front Immunol. 2025 Dec 19;16:1664444. doi: 10.3389/fimmu.2025.1664444 (PMC12757357; doi:10.3389/fimmu.2025.1664444)
Supplement: Supplementary file 1 [file DataSheet1.pdf]

Supplementary Material

Supplementary Table 1. Reagents and materials.

REAGENT or RESOURCE	Source	Cat#	Dilution	RRID
Antibodies				
Hu CD282 BUV615 11G7 50ug	Becton Dickinson	751484	1:40	AB_2875480
Mouse Anti-Human CD163 BUV395 MAC2-158 100 Tests	Becton Dickinson	568191	1:50	Not found
Hu CD206 (MMR) BV786 15-2 50ug	Becton Dickinson	751773	1:50	AB_2875749
Hu CCR2 (CD192) BV711 LS132.1D9 50ug	Becton Dickinson	747848	1:50	AB_2872310
Hu CD86 BV650 2331 (FUN-1) 100Tst	Becton Dickinson	563412	1:50	AB_2916294
Hu CD36 BV605 CB38 100Tst	Becton Dickinson	563518	1:50	AB_2738250
Mouse Anti-Human CD3 BV510 UCHT1 100 Tests	Becton Dickinson	568556	1:50	AB_2941961
Hu CD80 BV421 L307.4 100Tst	Becton Dickinson	564160	1:50	AB_2738632
Hu CD16 APC-H7 3G8 100Tst	Becton Dickinson	560195	1:50	AB_1645466

Mouse Anti-Human HLA-DR R718 L243 100 Tests	Becton Dickinson	568578	1:50	Not found
Hu TLR9 APC eB72-1665 100Tst	Becton Dickinson	560428	1:10	AB_1645622
Hu CD64 PE-Cy7 10.1 50Tst	Becton Dickinson	561191	1:50	AB_10611997
Hu CD184 PE-Cy5 12G5 100Tst	BIOLEGEND	306528	1:50	AB_2565994
Hu CD14 PE-CF594 MPHIP9 100Tst	Becton Dickinson	562335	1:50	AB_11153663
Hu TLR4 (CD284) PE TF901 100Tst	Becton Dickinson	564215	1:50	AB_2738674
Hu CD209 FITC DCN46 100Tst	Becton Dickinson	551264	1:50	AB_394122
Live/dead fixable Aqua dye	Thermo Scientific	L34957	1:50	Not found
Stimulus				
Pulmonaron PML	Sanofi-Aventis	S/N	$1x10^8$ CFU	Not found
IPI Suspensión Bacteriana	ASAC Pharmaceutical Immunology, ASAC S.A., México	S/N	$1x10^6$ CFU	Not found
M. bovis BCG	ATCC	35743	$1X10^6$ CFU	Not found

LPS Lipopolysaccharide from E. coli serotype 055:B5	Sigma-Aldrich	L2880	100ng/ml	Not found
Mtb H37Ra	ATCC	25177	$1x10^7$ CFU	Not found
Chemicals				
Lymphoprep	Axis Shield	18061		Not found
Monocyte isolation Kit	Miltenyi	130-050-201		AB_2665482
PBS 1x	Corning™Mediatech™	21-040-CV		Not found
RPMI-1640	Corning™Mediatech™	15-040-CV		Not found
Heat inactivated normal human serum	Valley Biomedical	HUMANSRMP-HI-1		Not found
Gentamicine	Lonza	GA-100		Not found
L-Glutamine	Lonza	BW17-605E		Not found
Brilliant Stain Buffer	Becton Dickinson	563794		Not found
RNeasy Microkit	Qiagen	74104		Not found
kit SuperScript First-Strand Synthesis System	Invitrogen	18091050		Not found
Cytometer Beads Array	Becton Dickinson	551811		AB_2868941
TaqMan Assays				
PFKP	Applied Biosystems	HS00737347_m1		Not found

HK2	Applied Biosystems	HS00606086_m1		Not found
GLUD1	Applied Biosystems	HS03989560_s1		Not found
GLS	Applied Biosystems	HS01014020_m1		Not found
CAMP	Applied Biosystems	HS00189038_m1		Not found
NPC2	Applied Biosystems	HS00197565_m1		Not found
MAP1LC3	Applied Biosystems	HS00797944_s1		Not found
ATG16L1	Applied Biosystems	HS00250530_m1		Not found
Software				
GraphPad Prism	Graphpad Software	https://www.graphpad.com		SCR_002798
Flowjo V10.10	Becton Dickinson	https://www.flowjo.com/flowjo/overview		SCR_008520
BioRender	bioRender	https://www.biorender.com/		SCR_018361

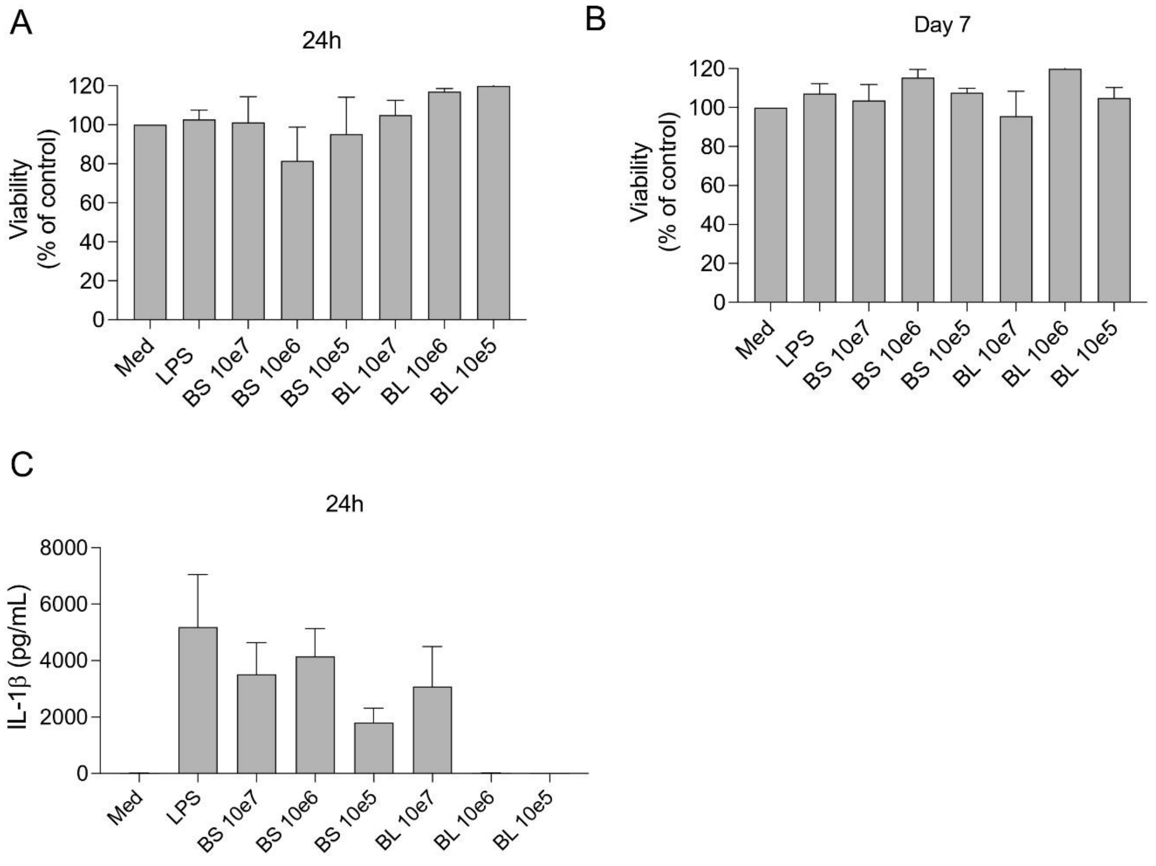

Supplementary Figure 1. BS and BL oral formulations are useful for *in vitro* assays. BS and BL were assayed to determine their safety and immunomodulatory capacity for *in vitro* cell cultures. Human primary monocytes were stimulated with different concentrations of BS and BL. (A, B) Different concentrations of BS and BL were used to determine the range of concentrations that were not toxic. Cell viability was measured at 24 h and day 7. (C) IL-1β production was measured at 24 h to determine the immunostimulatory capacity of BS and BL. Medium was used as a negative control, and LPS as a positive control. Depicted are means with SE, n=4.

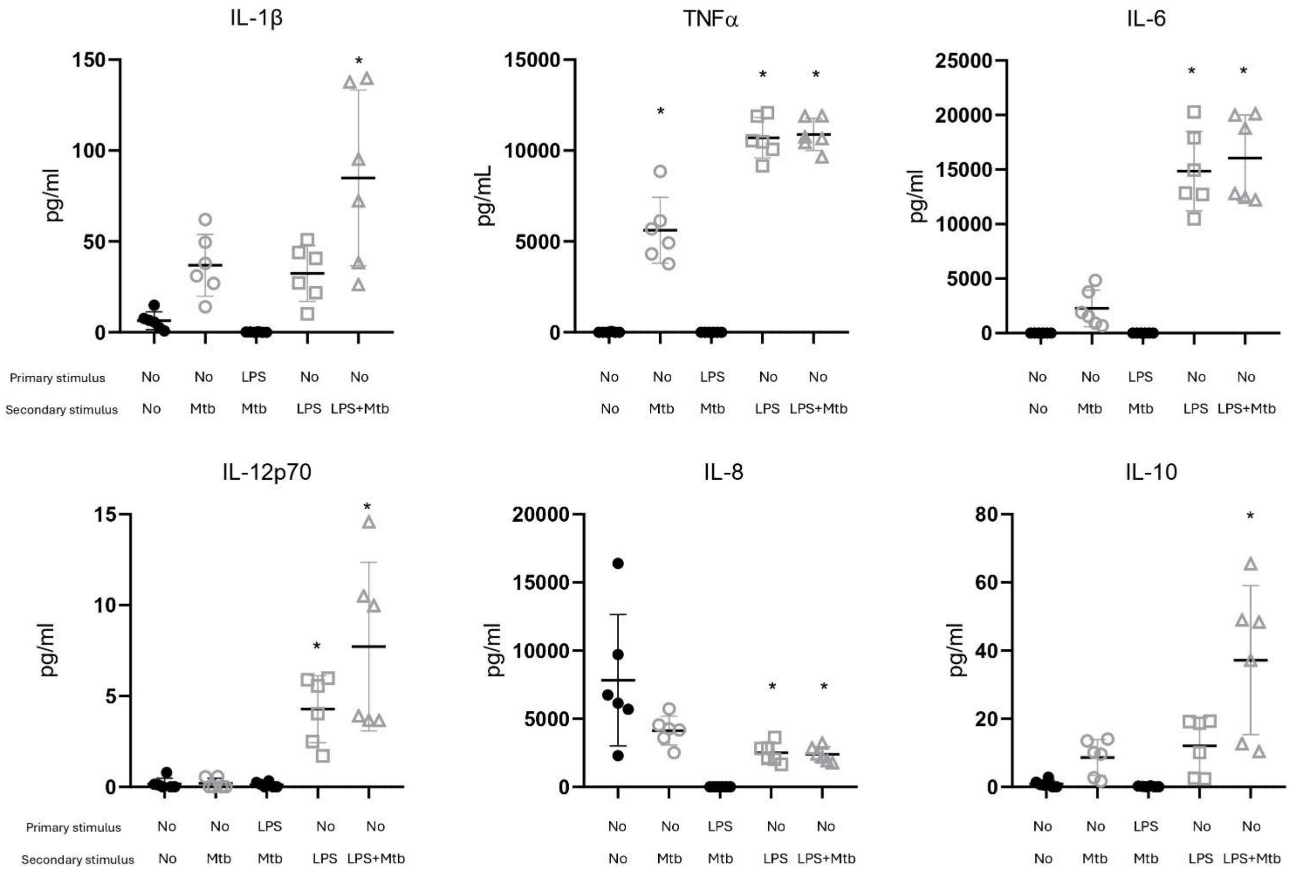

Supplementary Figure 2. LPS tolerizes macrophages, preventing them from responding rapidly to subsequent stimulation. LPS stimulated primary monocytes following the training protocol. Cytokines in supernatants of day 7 were measured using the CBA technology by flow cytometry. Depicted are individual results with means and SD. * $p<0.05$ compared to untrained, unstimulated macrophages. One-way ANOVA, followed by Dunnett's post hoc (n=6).

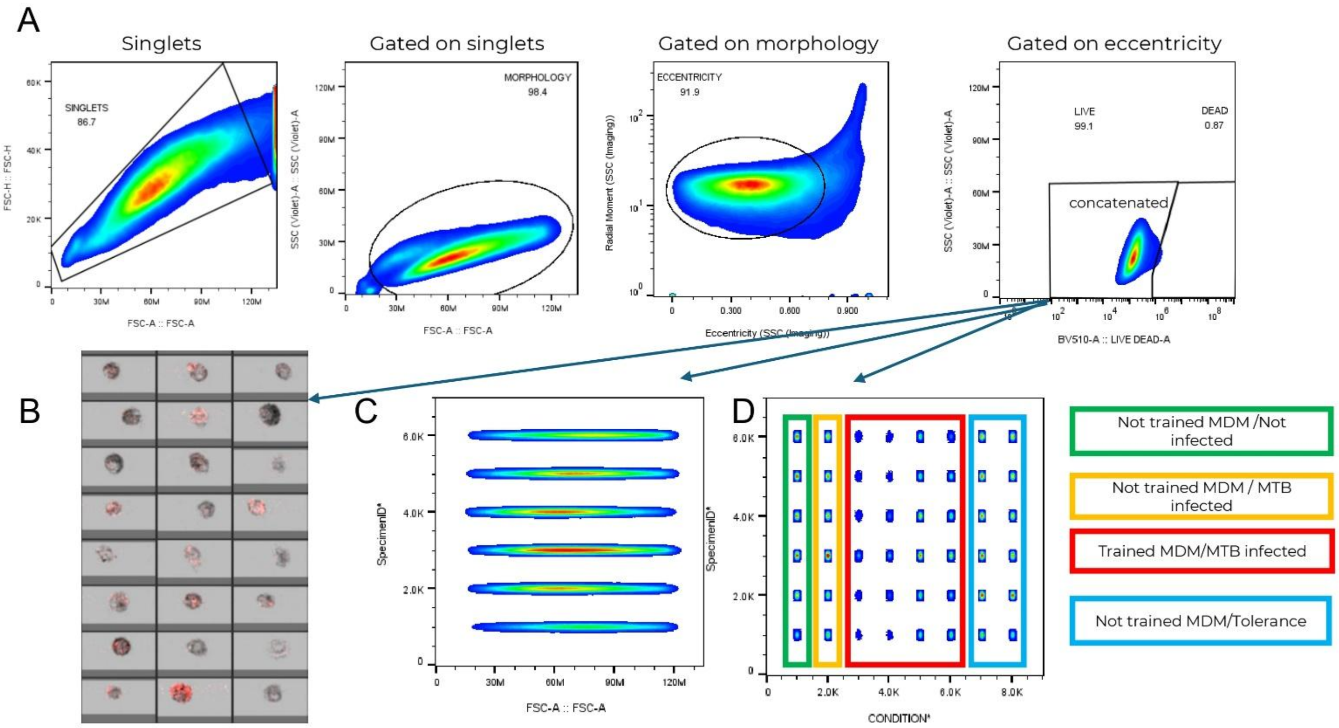

Supplementary Figure 3. Gating strategy for flow cytometry analyses. (A) We first gated singlets, then gated morphology by size and complexity, then eccentricity, followed by exclusion of dead cells and $CD3^+$ cells (detected in the same detector as dead cells). Live single macrophages of 6 individuals were concatenated into a single file. (B) The CellView window confirmed the single cell selection. (C) Specimen ID denotes the number of subjects that were concatenated. (D) The eight experimental conditions for the six subjects are displayed.

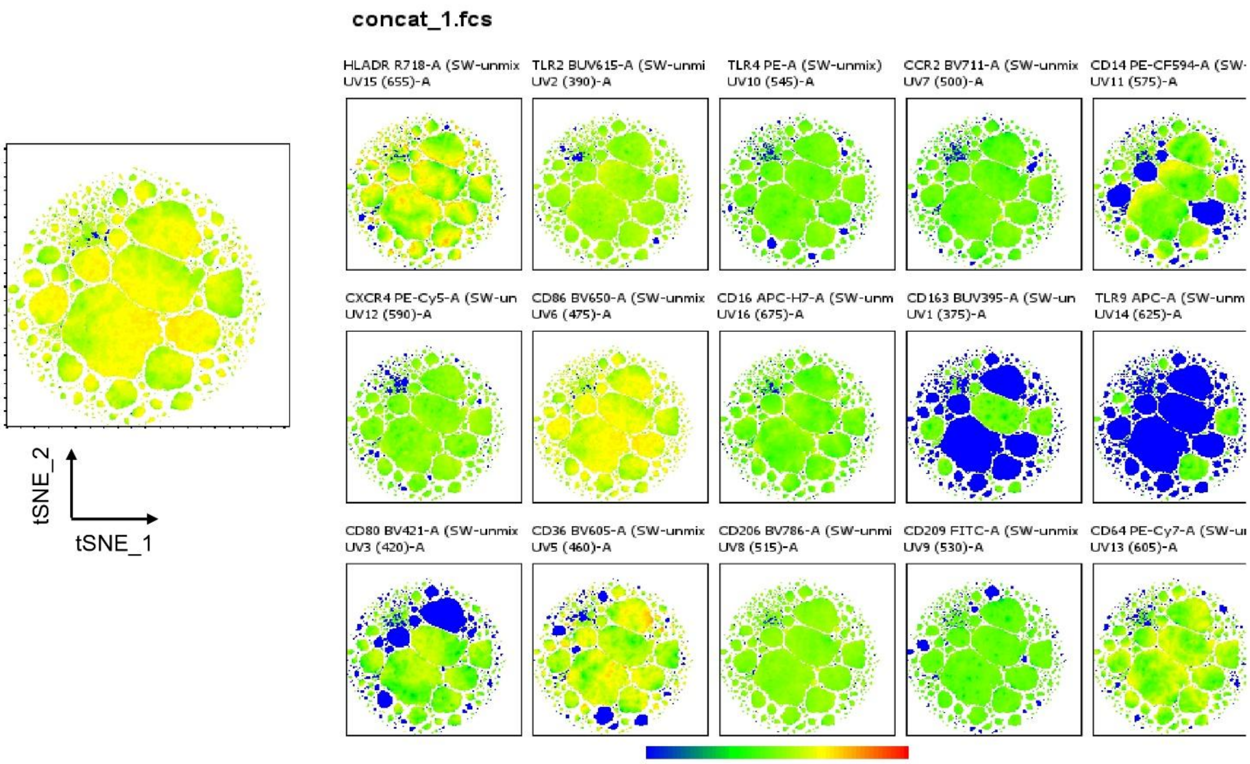

Supplementary Figure 4. Membrane markers individual expression. Macrophages were infected with *M. tuberculosis* (multiplicity of infection, MOI 5) for 24 hours. Cells were stained with antibodies anti CD80, CD86, TLR2, TLR4, HLA-DR, CD64, CCR2, CD14, CD16, CD206, CD209, CD163, CD36, CXCR4, and TLR9 to determine their expression by spectral flow cytometry. All live single macrophages from 6 individuals were concatenated (2,405,332 total events) and analyzed using dimensionality reduction via t-SNE.
